# Ameliorative Effects of a Polyphenolic Fraction of *Cinnamomum zeylanicum* L. Bark in Animal Models of Inflammation and Arthritis

**DOI:** 10.3797/scipharm.1301-16

**Published:** 2013-02-25

**Authors:** Badal Rathi, Subhash Bodhankar, V. Mohan, Prasad Thakurdesai

**Affiliations:** 1 Poona College of Pharmacy, Bharati Vidyapeeth Deemed University, Erandwane, Pune- 411 038, India.; 2 Indus Biotech Private Limited, 1, Rahul Residency, Plot Nos. 6 & 7, Off Salunke Vihar Road, Kondhwa, Pune-411 048, Maharashtra, India.

**Keywords:** Cinnamon bark, Antiinflammatory, Rheumatoid arthritis

## Abstract

Cinnamon bark (*Cinnamomum zeylanicum* Syn *C. verum*, family: Lauraceae) is one of the oldest traditional medicines for inflammatory- and pain-related disorders. The objective of the present study was to evaluate the efficacy of the polyphenol fraction from *Cinnamomum zeylanicum* bark (CPP) in animal models of inflammation and rheumatoid arthritis. Dose-response studies of CPP (50, 100, and 200 mg/kg) used in a separate set of *in vivo* experiments were conducted in acute (carrageenan-induced rat paw edema), subacute (cotton pellet-induced granuloma), and sub-chronic (AIA, adjuvant-induced established polyarthrtis) models of inflammation in rats and the acetic acid-induced writhing model of pain in mice. Effects of CPP on cytokine (IL-2, IL-4, and IFNγ) release from Concanavalin (ConA)-stimulated lymphocytes were also evaluated *in vitro*. CPP showed a strong and dose-dependent reduction in paw volume, weight loss reversal effects against carrageenan-induced paw edema, and cotton pellet-induced granuloma models in rats. CPP (200 mg/kg p.o. for 10 days) showed a significant reduction in elevated serum TNF-α concentration without causing gastric ulcerogenicity in the AIA model in rats. CPP also demonstrated mild analgesic effects during acute treatment as evidenced by the reduction in the writhing and paw withdrawal threshold of the inflamed rat paw during the acetic acid-induced writhing model and Randall-Selitto test. CPP was found to inhibit cytokine (IL-2, IL-4, and IFNγ) release from ConA-stimulated lymphocytes *in vitro*. In conclusion, CPP demonstrated prominent action in animal models of inflammation and arthritis and therefore can be considered as a potential anti-rheumatic agent with disease-modifying action.

## Introduction

Rheumatoid arthritis (RA) is a chronic, inflammatory autoimmune disease affecting freely movable joints, such as hand, knee, and shoulder joints. The symptoms of active RA include pain, swelling, morning stiffness, warmth, redness, and limited functioning of the joints. The systemic ramifications of the disease, apart from morbidity and mortality, include cardiopathy, nephropathy, vasculopathy, pulmonary, and cutaneous disorders [1]. Although the cause of rheumatoid arthritis is unknown, autoimmunity plays a pivotal role in both its chronicity and progression, and RA is considered to be a systemic autoimmune disease. Existing treatment therapies for RA usually focus on anti-inflammatory activity. They help to manage the process of inflammation and thus may also help in the repair process. Medications like nonsteroidal anti-inflammatory drugs (NSAIDs), corticosteroids, and analgesics are used to suppress symptoms, while disease-modifying anti-rheumatic drugs (DMARDs) and biological response modifiers are often required to inhibit or halt the underlying immune process and prevent long-term damage. Therefore, the search for safer drugs for the management of rheumatoid arthritis for chronic use is still on.

Cinnamon bark (*Cinnamomum zeylanicum* Syn *C. verum*, family: Lauraceae) is one of the oldest herbal medicines mentioned in many traditional texts for inflammation [2] and pain related to disorders such as enteralgia (acute intestinal pain), bronchitis, and rheumatism [3]. It is native to Sri Lanka Mayanmar (Burma) and the southern coastal strip of India. In Chinese traditional medicine, cinnamon is indicated as an analgesic and antipyretic against colds, fever, headache, myalgia (mascular pain), arthralgia (arthritic pain), and amenorrhea (failure of menstruation). In Indian traditional literature including Ayurveda and ‘Materia Medica of India and their Therapeutics’, many other valuable actions are attributed to cinnamon bark and oil [4].

Many scientific pharmacological investigations have also reported on the anti-inflammatory potential of bark of many species of cinnamon [3, 5, 6]. The anti-inflammatory action of the Japanese species Cinnamomum seiboldii [7] and Cinnamomi cortex [8] has been attributed to a series of tannins. An herbal ophthalmic medicament called Ophthacare, which contains 0.5% cinnamon, was tested for its anti-inflammatory activity on ocular inflammation in rabbits and found to be effective [9]. The antinoiceptive activity (analgesic) [10] and antipyretic (fever-reducing) activity of cinnamon (*C. verum*) bark [7] were also reported.

Another important activity is the immunomodulatory effects exerted by cinnamon. An interesting fact about cinnamon is that it can act both as an immunostimulant and an immunosuppressant depending on the species and dose [11]. *In vitro* inhibitory activity against the complement formation has been documented for cinnamon cortex and cinnamon oil [11]. The extract of cinnamon bark is reported to have anticomplementary activity [12] and immunosuppressive activity [13, 14]. Cinnamon bark’s potential for relieving inflammation and pain, and enhancing the immune system, makes it a good candidate as an antiarthritic agent.

Furthermore, cinnamon polyphenol extract (CPE) from the cinnamon bark of various varieties has shown potential for the management of certain human health conditions. Procyanidins or condensed tannins are flavonoid oligomers whose building blocks are (+)-catechin and (−)-epicatechin. They are oligomeric end products of the flavonoid biosynthetic pathway and are now identified and recognised for their favourable effects in human beings. Based on the linkage between the successive monomeric units, procyanidins are classified as Types A, B, or C procyanidine polyphenols.

Recently, the immunomodulatory effect of the water extract of cinnamon on anti-CD3-induced cytokine responses and p38, JNK, ERK1/2, and STAT4 activation [13] has been shown. CPE is known to affect immune responses by regulating anti- and pro-inflammatory and GLUT gene expression as seen in an *in vitro* study on mouse RAW264.7 macrophages [15]. However, a functional outcome of these effects in an animal model of rheumatoid arthritis has not been investigated.

The objective of the present work is aimed to investigate the functional effects and probable mechanism of action of polyphenols isolated from *Cinnamomum zeylanicum* bark (CPP) in an animal model of acute inflammation and established rheumatoid arthritis in rats. CPP was tested against animal models of acute (carrageenan-induced), subacute (cotton pallet granuloma), and chronic (Freund’s complete adjuvant, FCA, induced established poly arthritis) models of inflammation. CPP was also tested for its potential analgesic activity against inflammaotoy pain and ulcerogenicity.

## Results and Discussion

### Characterization of CPP

**Fig. 1:**
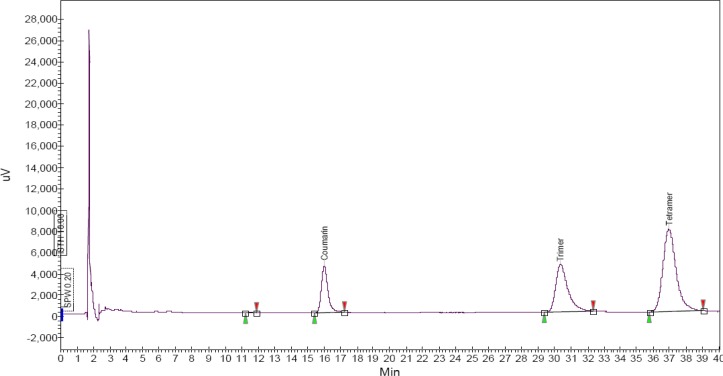
HPLC spectra of CPP at 280 nm.

As an initial chemical characterization of the CPP, total phenolic content was determined and found to be 860 mg of gallic acid equivalant (GAE) per g. As an additional characterization, an HPLC study was performed. The HPLC spectra showed the presence of polyphenols with a similar absorption pattern to procyanidines with two bands of maximum absorption around a retention time of 30 to 38 min, which matches with the trimer and tetramer of proanthocyanidins polyphenols as reported earlier [16] and suggests the predominance of polyphenolic compounds in CPP (Fig. 1).

### Effect of CPP against carrageenan induced rat paw edema in rats

As shown in Table 1, subplanter administration of carrageenan caused an increase in the paw volumes of rats (an increase of 2.63 ml within 3 hours) which indicates edema formation (inflammation) in rat paws. Acute oral administration of celecoxib (10 mg/kg) prevented the edema formation significantly (P < 0.001) and showed an increase in the paw volume by 1.42 ml (45.79% inhibition) at 3 hours. Acute oral administration of CPP showed dose-dependent inhibition of carrageenan-induced edema (21.83, 33.19, and 39.97% inhibition) at 50, 100, and 200 mg/kg, p.o., respectively, at 3 h.

None of the treatments showed significant inhibition of paw edema at 1 h after carrageenan administration. However, at 2 h, CPP showed significant inhibition of paw edema at a dose of 200 mg/kg (37.27% inhibition).

### Effect of CPP on pain threshold during the Randall-Selitto test in rats

The pain threshold was measured mechanically at 3 h and pressure levels at which rats elicited the response of struggling, squealing, or vocalization were taken as the pain threshold. Vehicle control rats showed a PWT of 66 g. Celecoxib (10 mg/kg, p.o) increased paw withdrawal latency (PWT) by 36.4%, whereas CPP (100 and 200 mg/kg, p.o.) increased PWT by 16.66 and 20.73%, respectively. However, CPP (50 mg/kg, p.o.) did not increase PWT significantly as compared with the vehicle-treated rats. Thus, CPP demonstrated mild analgesic activity on the pain responses elicited by the mechanical stimulus.

### Effect of CPP in cotton pellet granuloma in rats

Implantation of cotton pellets produced granuloma formation, which was measured by weighing the dried pellet after 7 days post-implantation. In the vehicle-treated rats, the mean cotton pellet weight was increased by 123% (from 20 g to 44.67 g). After seven days of treatment with celecoxib (10 mg/kg/day) and CPP (50, 100, and 200 mg/kg/day), the dry weights of the cotton pellet granuloma were found to reduce by 43.51%, 27.31%, 34.40%, and 38.92%, respectively, which was significant as compared with the weights of granuloma tissues in the vehicle control rats (Table 2).

### Effect of CPP against acetic acid-induced writhing in rats

The mean number of writhes induced by the intraperitoneal administration of acetic acid (0.1 ml, 0.6% v/v) was 29.17 in the vehicle control group which confirms the induction of abdominal pain (Table 2). The mean numbers of writhes were significantly reduced in animals pretreated orally with celecoxib (13.83 writhes, 52.59% reduction) and CPP (22 writhes, 24.58% reduction). However, CPP did not exhibit a significant reduction in writhing at an acute oral dose of 50 mg/kg or 100 mg/kg (Table 2).

### Effect of CPP against FCA-induced established polyarthritis (AIA model)

#### Effect on Body weight in the AIA model

Immunization of rats with FCA produced a gradual and significant (P < 0.001) decrease in the body weight of rats as observed on day-12 of the study in all rats (Table 3). The decrease was sustained in adjuvant-induced arthritic rats treated with the vehicle (AIA control). Nine days of daily oral administration (day-12 to day-21) of CPP (50, 100, and 200 mg/kg/day) and celecoxib (10 mg/kg/day) caused a dose-dependent and significant (P < 0.001) reversal of the FCA-induced body weight decline as seen on day-21.

#### Effect on paw edema in the AIA model

The immunization of rats by a subplanter injection of FCA induced a local inflammatory reaction in the injected paw (primary response) and a secondary response in the non-injected (contralateral) paw in 12 days post-immunization (Table 3). The significant increase in paw volumes was observed both in the injected and non-injected (contralateral) paws on day-12 of the study in all rats (Table 3) confirming the induction of arthritis in rats. Oral treatment of CPP (50, 100, and 200 mg/kg/day) and celcoxib (10 mg/kg/day) from day-12 to day-21 (9 days), brought upon a dose-dependent and significant (P < 0.001) reduction in the paw volumes in FCA-injected paws (Table 3). On the other hand, contralateral paw volumes were significantly reduced only by CPP (200 mg/kg/day for 9 days) treatment (P < 0.001) (Table 3). Celecoxib (10 mg/kg) and CPP (50 and 100 mg/kg) did not show a significant reduction in contralateral paw volumes on day-21 post-immunization (Table 3).

### Effect on serum TNFα levels in the AIA model

***Administration of FCA into the sub-plantar region of rats*** significantly (P < 0.001) increased the serum TNFα concentration on day-12 in all the rats (Table 3). The serum TNF-α concentration continued to be at higher levels in the adjuvant-induced arthritis control (AIA) group as seen on day 21 (Table 3). Serum TNF-α concentration was significantly (P < 0.001) decreased on day 21 in rats treated with CPP (200 mg/kg/day) and celecoxib (10 mg/kg/day) for 9 days as compared to corresponding levels on day-12 (Table 3).

#### Effect on gastric mucosa in the AIA model

**Fig. 2:**
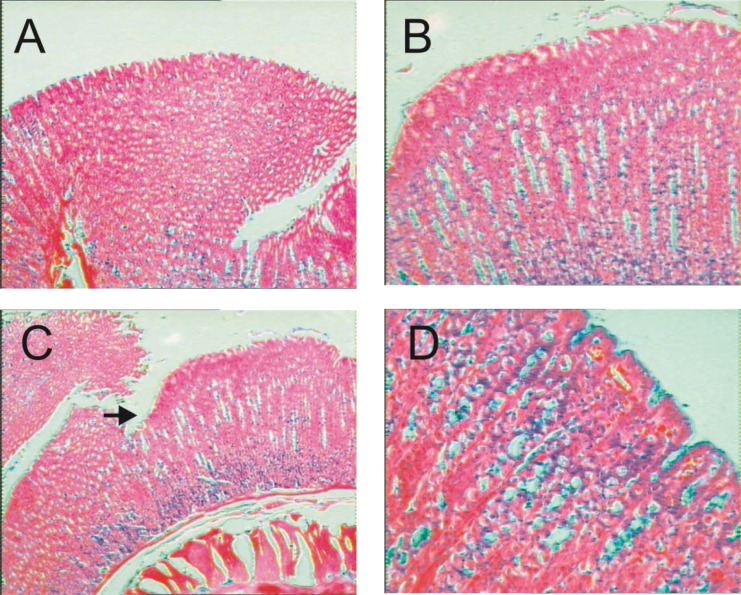
Photomicrograph of sections of stomach on Day 21 of the study. The figures show sections of stomach of rats with absence of arthritis (A) and FCA-induced arthritis-induced rats with 10-day treatment of vehicle (distilled water) (B), celecoxib (10 mg/kg, p.o.) (C) and CPP (200 mg/kg, p.o.). Arrow indicates erosion with hemorrhage at the superficial part of the mucosa. Stained with hematoxylin and eosin (H&E) X 200.

The gastric mucosal superficial layer of sections of stomach samples of rats removed from normal non-arthritic (Figure 2A) rats was found to be smooth. The mucosal epithelial layer of the stomach sections of FCA-induced arthritic rats after subacute treatment with the vehicle (Figure 2B) and CPP at oral dose of 200 mg/kg (Figure 2D) were also found to be normal and smooth with mild capillary dilation. However, the superficial layer of the gastric mucosa of rats treated with the subacute treatment of celecoxib (10 mg/kg, p.o.) had multiple areas of erosions and hemorrhage (Figure 2C).

### Effect of CPP on Concanavalin (Con-A)stimulated lymphocyte culture in vitro

The levels of IL2, IL4, and IFNγ in normal lymphocytes (non-stimulated) were 1.94, 25.02, and 20.41 pg/ml, respectively (Table 4). The ConA-stimulated proliferation of the splenic lymphocyte culture showed a significant (P < 0.001) increase in cytokine expression (IL-2, IL-4, and IFNγ levels were 589, 114.75, and 277.74 pg/ml, respectively). CPP treatment brought about a significant (P < 0.001) reduction in IL-2, IL-4, and IFNγ levels (Table 4) of ConA-stimulated lymphocytes and showed IL-2, IL-4, and IFNγ levels of 10.27, 41.22, and 21.98 pg/ml respectively.

#### Discussion

Although NSAIDs, DMARDs, and corticosteroids appear to be highly efficient drugs in the treatment of rheumatoid arthritis, they may cause side effects that can range in severity from mild to serious. The major adverse drug reactions (ADRs) associated with NSAIDs are gastrointestinal (ulceration or bleeding) with effects on other systems. During their clinical use, gastrointestinal toxicities especially as upper gastrointestinal adverse events such as perforation, ulceration, and bleeding are reported in about 20% of patients taking long-term NSAIDs which is a major clinical limitation [17]. Besides, some NSAIDs were recently withdrawn from the market because of the risk of heart attacks and stroke, and some other NSAIDs contained a ‘Black Box’ Warning in the package-inserts, addressing the risk of suffering from heart attacks and/or stroke [18, 19]. On the other hand, other arthritis therapies such as DMARDs and biological agents had risks of immune suppression and serious infection, respectively, with long-term usage.

Proanthocyanidins (procyanidin, oligomeric proanthocyanidin (OPC), leukocyanidin, leucoanthocyanin, and condensed tannins) are a class of flavanols. The cinnamon polyphenol extract (CPE) is water-soluble and can be standardized to proanthocyanidins [20]. Proanthocyanidins are essentially polymer chains of flavonoids such as catechins [21]. The anti-inflammatory action of many species of cinnamon has been attributed to the polyphenolic component such as tannins [7] and procyanidins [21]. Therefore, we have successfully prepared the standardized polyphenol fraction from the hydroalcoholic extract of *Cinnamomum zylanicum* bark (labelled as CPP) and evaluated it against animal models of inflammation and arthritis. Total phenolic content (TPC) of CPP was also determined by the well-established Folin-Ciocalteu reagent method [22] and was found to be 860 mg GAE/g in the present study.

The carrageenan-induced rat paw edema is an *in vivo* test for the evaluation of acute inflammatory activity. The recognition of different mediators for different phases of the carrageenan-induced edema has important implications for interpreting the anti-inflammatory effects of the drugs. The development of such edema in the rat paw is a biphasic event. The initial phase of the edema has been attributed to the release of histamine and serotonin which is then maintained during the plateau phase because of kinin-like substances [23]. Further, involvement of histamine and serotonin with other mediators in the pathogenesis of rheumatoid arthritis was also reported by many workers [24–26]. Effectiveness of the test compounds in the first hour after carrageenan injection is indicative of their antihistaminic and/or anti-serotonin action. Second, the accelerating phase of carrageenan edema is attributed to the release of prostaglandins [27].

Our test compound, CPP was not effective in the first hour of treatment but was found to be effective in the third hour at 100 mg/kg and at higher doses, suggesting prostaglandin inhibition as a possible mechanism of the action effect (Table 1). Tissue granulation is one of the distinctive features of inflammation, which is composed of a marked infiltration of macrophages and neovascularisation. This condition can be induced in rats by subcutaneous implantation of a sterile cotton pellet. The implanted material induces the host’s inflammatory response and modulates the release of inflammatory mediators which finally leads to tissue proliferation and granuloma formation [28]. The cotton pellet-induced granuloma is closely related to the formation of antibodies and the resultant inflammation involves the infiltration of macrophages and neutrophils, and the proliferation of fibroblasts. The multiplications of small blood vessels as well as the proliferation of fibroblasts are the characteristic features at the repair phase of inflammation [29]. Such proliferating cells penetrate the exudates, producing a highly vascularized reddened mass known as granulation tissue [30]. In our study, CPP showed strong (P < 0.001) and dose-dependent inhibition of cotton pellet granuloma and is indicative of its potential to hasten the repair phase of inflammation.

Analgesia is an important ancillary property of all anti-inflammory agents. Most of the anti-inflammatory drugs increased the pain threshold in various animal models [31]. This is natural because many endogenous chemical mediators of inflammation play a part in generating pain impulses (for example histamine, serotonin, prostaglandins), and some other mediators such as bradykinin and cytokines are involved in the prolongation of the sensation of the pain [32].

The syndrome of writhing—lengthwise stretches of the torso accompanied (usually) by concave arching of the back—produced by acetic acid, was sensitive and a well-accepted method for the evaluation of mild analgesic nonsteroidal anti-inflammatory compounds [33–35]. The writhing test is also largely employed as an assay of visceral nociception, which represents a major clinical problem [36]. Writhing, thought to be reflexive in nature, is produced by a caudally directed wave of abdominal wall muscle constrictions and elongations, and is often followed by a characteristic hind limb extension. Acetic acid stimulates several inflammatory mediators such as cytokines and eicosanoids, which indirectly sensitizes the pain nerve endings and causes the abdominal constriction [37]. In the present study, intraperitoneal administration of acetic acid induced a severe writhing response, whereas CPP showed a mild reduction in acetic acid-induced writhing at 200 mg/kg, p.o.

The pain threshold of the inflamed edematous right hind rat paw was subjected to constant force, and examined by the Randall-Selitto assay method [38]. In the present study, CPP could show only mild reduction (20%) in the pain latency threshold (PWT) of carrageenan-induced rat paw edema as shown in the Randall-Selitto assay (Table 1). The mild analgesic effect shown by CPP is indicative of stimulus propagation or cytokine blockage in the pain nervous fibers as the basis of the analgesic effect.

Adjuvant-induced arthritis (AIA) in rats is commonly used to evaluate compounds that might be of potential use as drugs for the treatment of rheumatoid arthritis and other chronic inflammatory conditions. Injection of Freund’s adjuvant suspended in mineral oil (Freund’s complete adjuvant, FCA) into rats produces an immune reaction that characteristically involves inflammatory destruction of cartilage and bone of the distal joints with concomitant swelling of surrounding tissues. FCA induced a series of cellular events leading to T-cell activation and polyarthritis [39].

AIA is a rather aggressive and monophasic form of arthritis; usually, the disease is quite severe and finally leads to complete ankylosis and permanent joint deformations. The progression of arthritis was confirmed in our study by the increase in edema (paw volume). The diseases progressed until day-12 and then became quiescent. CPP halted the progression which was evident by a significant decrease shown in the paw volume (Table 3).

The inflammation associated with AIA is dependent on prostaglandin E2 (PGE2) generated by cyclooxygenases (COXs) [40, 41]. Besides, the role of cytokines like TNF-α and IL-1 has also been implicated in this model [42]. In the present study, CPP treatment for 10 days reduced paw volumes of arthritic paws in rats. Therefore, the anti-inflammatory action of CPP can be attributed to prostaglandin and/or cytokine inhibition. These results are also in line with reports that the anti-inflammatory action of cinnamon bark has been attributed to polyphenolic components such as tannins [7] and procyanidines [21]. The inhibition of lipid peroxidation, capillary permeability and fragility, and enzymes such as phospholipase A2, cyclooxygenase, and lipoxygenase was reported as the alleged mechanism for cinnamon tannins and polyphenols [7, 21]. However, the absence of the analgesic effects of CPP as indicated in the acetic acid-induced writhing model (Table 2) suggested cytokine inhibition rather than prostaglandin inhibition as the potential mechanism for the anti-inflammatory effect of CPP.

One of the most prominent and serious side effects of NSAIDs is the occurrence of gastrointestinal damage (ulcerogenicity). In the present study, for subacute treatment, CPP (200 mg/kg p.o. for 10 days) did not cause ulcers in the established AIA in rats (Figure 2). Further, cinnamon bark extract and polyphenols have been shown to possess cytokine inhibitory effects *in vitro*[15, 43]. An *in vitro* pre-challenge with cinnamon extract is reported to suppress lipopolysaccharide (LPS)-induced cytokine expression [44]. The absence of ulcerogenicity, and mild analgesic potential with good anti-inflammatory activity, is in line with the view that CPP has disease-modifying potential. In addition, cytokine inhibition emerges as the probable mechanism for CPP which is well-supported by its cachexia-reducing effects shown in established AIA-induced polyarthritis in rats in the present study.

Cytokines are known to regulate several events in immune response including cell cooperation, proliferation, differentiation, and death. Research has also suggested that skeletal muscle protein loss is dependent upon the signaling activities of both TNF-α and IFN-γ, and that nuclear factor kappa B (NFκ_B_) activity is needed for these cytokines to induce muscle damage [45]. In chronic inflammation, an activated immune system can release a series of pro-inflammatory cytokines, including tumor necrosis factor- (TNFα). RA progression is associated with an imbalance of Th1/Th2 and overproduction of antigen-specific immunoglobulin [46]. Several cytokines (TNF-α, IL-1, IL-6, and GM-CSF) and activated B cells, T-cells, and antigen-presenting cells play a crucial role in RA joint inflammation. The efficacy of anti-TNFα and anti-IL-1 agents in the treatment of RA patients is established [47, 48]. In the present investigation, the effect of CPP on serum TNFα release from FCA-induced arthritic rats at a dose of 200 mg/kg was estimated. In our study, AIA rats showed a significant increase in serum TNFα levels on day 12. CPP treatment (for 10 days) showed a significant reduction in elevated TNFα levels in AIA rats on day 21 of the study (Table 3). AIA in rats is also a useful model of inflammatory cachexia that mimics the human pathophysiology in many important ways [49]. Secondary reactions in AIA are characterized by immune responses, inflammation, and severe weight loss (cachexia). In our study, the test compound CPP significantly protected rats from AIA-induced cachexia (Table 3). Cachexia associated with RA persists even after joint inflammation improves [50]. Further, rheumatoid cachexia leads to muscle weakness and a loss of functional capacity, and is believed to accelerate morbidity and mortality in rheumatoid arthritis [51]. Excess production of the inflammatory cytokines is known to have a definite role in the development of rheumatoid cachexia. TNFα is probably the central mediator of muscle wasting in RA, and is known to act synergistically with interleukin-1β to promote cachexia [51].

Cytokines elaborated by activated T-cells play a major role in the regulation of immune responses. When environmental substances (molecules or microbes) gain access to the body, these substances (termed antigens) are recognized as foreign by antigen receptors that are expressed on the surface of lymphocytes. In an attempt to further understanding of the role of cytokines in the mechanism of CPP, we have analyzed T-cell-derived cytokines in the spleen lymphocyte supernatant isolated from mice. The proliferation response of T-cells to a polyclonal mitogen, ConA, in culture supernatant and their cytokine secretion profiles are well-documented [52]. These cytokines are normally produced by the body during an immune response [53]. In our study, the test compound CPP was found to inhibit cytokines (IL-2, IL-4, and IFN γ) in ConA-stimulated spleen cell culture supernatant (Table 4). IL-2 is necessary for the development of T-cell immunologic memory, one of the unique characteristics of the immune system, which depends upon the expansion of the number and function of antigen-selected T-cell clones. IL-2 is also necessary during T-cell development in the thymus for the maturation of regulatory T-cells (Tregs) [54] which results in autoimmunity. IL-4 is an important immune cytokine which regulates the function of lymphocytes and macrophages [55]. Several lines of evidence exist confirming the role of Th2 cell activation and production of cytokines such as IL-4 in RA and other autoimmune diseases [56–58]. Further, circulating IFN γ can be detected in patients with autoimmune diseases like RA [59]. These results suggest the inhibitory effects of CPP on lymphocyte proliferation and the reduction of autoimmunity as a probable basis for the disease-modifying anti-rheumatic effects of CPP in RA.

Taken together, our results indicated cytokine inhibition as the probable mechanism of action of CPP against rheumatoid arthritis. Further, the lack of analgesic action and ulcerogenicity pointed to CPP as a disease-modifying anti-rheumatic drug (DMARD). DMARDs suppress the rheumatoid process and bring about a remission, but do not have non-specific anti-inflammatory or analgesic action. CPP can provide added advantages such as lack of gastric and cardiovascular side effects. Cinnamon bark extract had been reported to inhibit gastric secretions [11] and irradiate *H. pylori*, a bacteria known to exacerbate gastric acidity and ulcers [60, 61]. Cinnamon bark was also expected to have protective effects on the cardiovascular system due to its potent antioxidant capacity [62–66].

In conclusion, CPP showed good ameliorative effects in animal models of inflammation and rheumatoid arthritis and can be explored as a DMARD.

## Experimental

### Animals

Male Wistar rats (130–200 g) or Swiss albino mice (20–25 g) were obtained from the National Toxicology Centre, Pune, India. The animals were housed in polypropylene cages at a temperature of 24 ± 1 °C in 12h:12h dark–light cycle, with free access to standard pellet feed (Chakan Oil Mill, India) and filtered water. All experiments were carried out between 08:00 h and 17:00 h in a quiet laboratory at an ambient temperature. The research protocol (No: CPCSEA/48/08) was approved by the Institutional Animal Ethics Committee (IAEC) and as per Indian norms laid down by the Committee for the Purpose of Control and Supervision of Experimental Animals (CPCSEA), New Delhi.

### Chemicals

For the in vivo studies, carrageenan and FCA (Sigma-Aldrich, St.Louis MO, USA), acetic acid (Pure Chem. Limited Ltd, Pune, India), carboxymethylcellulose (Qualigens, Mumbai, India), anaesthetic ether (Batch No. 30, TKM Pharma, Hyderabad), and the BD-OptEIA kit for TNF α estimation (BD Pharmingen, U.S.A) were purchased from the respective vendors. All other chemicals used were of analytical grade.

For the in vitro studies, fetal calf serum (FCS) and culture media were purchased from GIBCO BRL (Grand Islan, NY, USA). RPMI1640 (Sigma-Aldrich, St.Louis MO, USA) and Concanavalin A (ConA, Calbiochem, USA) were purchased from the respective vendors. Starch, ethanol, and all other chemicals were purchased from reputed local manufacturers. All plasticwares that were used for the in vitro studies were purchased either from Nalgene Nunc (Naperville, IL, USA) or Falcon (Becton Dickinson Labware, USA).

### Extraction and characterization of CPP from Cinnamon bark

Cinnamon bark (1000 g) pieces were dried and pulverized to make powder (16 mesh). The powder was extracted with 6 L of a 95% ethanol-water mixture over a period of 10 h with the help of an extractor having a perforated bottom sieve of the 200 mesh size. The bottom eluent was recycled repeatedly. The eluent was collected and concentrated at low temperature to get a paste. The paste was dissolved in 2000 ml of distilled water and the pH was adjusted to 3 with phosphoric acid and insolubles were filtered. The clear filtrate was passed through a layer of activated neutral alumina column (comprising a dry charge of 400 ml of alumina). The adsorbed material on the column was eluted with ethyl alcohol, and concentrated to get powder of CPP (also known as IND02) (Yield: 28 g). The obtained compound, CPP, was charactrized by HPLC and the total phenolic content was determined.

### Determination of Total Phenolic Content (TPC) of CPP

Total phenolic content was determined with the Folin-Ciocalteu reagent according to a procedure described by Singleton and Rossi [22]. An aliquot of 1 ml of the extract solution in methanol was mixed with 1 ml of the Folin-Ciocalteu reagent (previously diluted with water 1:1 v/v), 3 ml of saturated sodium carbonate (Na_2_CO_3_) solution (about 75 g/l), and 4 ml of water. The mixture was allowed to stand at room temperature for 25 min and was then centrifuged at 5000 rpm for 10 min. Absorbance of the supernatant was measured at 755 nm using a spectrophotometer (Shimadzu). Gallic acid was used as a reference standard, and the results were expressed as milligram gallic acid equivalent (mg GAE)/g dry weight of CPP.

### Anti-inflammatory activity against carrageenan-induced rat paw edema

Anti-inflammatory activity was evaluated on the basis of the inhibition of the carrageenan-induced hind paw edema [67, 68]. Each rat in the experimental group (six rats each) was treated orally with 50, 100, and 200 mg/kg body weight of CPP, 1 h before subplanter injection of the edematogenic agent, carrageenan (0.1 ml of a 1% suspension prepared in 0.9% NaCl) to the left hind paw of rats with a 26 gauge needle. One group of rats was treated orally with 10 mg/kg body weight of celecoxib as a standard drug. The separate vehicle control group was also maintained. Paw volume was measured immediately after the carrageenan injection (time 0) and at 1, 2, 3, and 24 h using a plethysmometer (Model 7150, Ugo Basile, Italy). The mean difference in paw volume (ml) was calculated with baseline (0 h) values for each rat. Percent (%) inhibition of paw volume was calculated with a reported formula [69] from the mean difference in mean paw volume (ml.) in treated (Vt) and control (Vc) groups according to the equation: % inhibition = (Vt/Vc)*100.

### Analgesic activity in the Randall-Selitto assay against carrageenan-induced rat paw edema

The pain stimulus of each rat was measured at the 3rd h after the carageenan-injected paw with the help of the Randall-Selitto analgesymeter (Ugo basile Model 7200) by the method reported earlier [38]. The apparatus was set up to apply a force of 0–500 g increasing from zero. The paw withdrawal threshold (PWT) was taken at the point at which the rat started to react (vocalized or struggled). The rats that did not attempt to remove their paws or vocalize before the 5000 g point (cut-off pressure) were discarded. Paw withdrawal threshold (PWT) was considered as a measure of pain latency. The percent increase in PWT was calculated as compared with the PWT of the vehicle control rats.

### Anti-inflammatory activity against cotton pellet granuloma

Subcutaneous implantation of pellets of compressed cotton provokes foreign body granuloma [70]. Male Wistar rats (175 to 200 g) were divided randomly in six groups with six rats in each group. The rats were fasted for 6 h before oral administration of the test compounds. Group 1 was the control group, Group 2 was the vehicle (distilled water)-treated; group 3 was celecoxib-treated (10 mg/kg p.o.) and Groups 4, 5, and 6 were CPP (50,100, and 200 mg/kg)-treated groups, respectively. Cotton rolls were cut and made into pellets weighing 20 mg each and sterilized in an autoclave at 100 °C for 30 min. Four individual pellets were inserted in each ether-anesthetized animal by making small subcutaneous incisions in both axillae and groin regions. The incisions were sutured by sterile catgut. After recovery from anesthesia, the animals were placed in individual cages and treated orally with test drugs for seven days starting from the day of cotton pellet implantation. The animals were anaesthetized again on the 8th day and cotton pellets were removed surgically, freed from extraneous tissue, incubated at 37 °C for 24 h, and dried at 55 °C to constant weight. The weight of the cotton pellet before implantation was subtracted from the weight of the dried dissected pellets. Thus any increment in the dry weight of the pellets was taken as a measure for granuloma formation. The mean weight was calculated for pellets from the group of rats receiving drugs and compared with the mean values for the control.

### Analgesic activity against acetic acid-induced writhing in mice

Male Swiss albino mice (20–25 gm) were divided into the following groups containing six animals in each group. Rats form Group I were administered orally with the vehicle (distilled water) and called the vehicle control. Rats from Group II were administered with celecoxib (10 mg/kg, p.o.). To rats of Group III, IV, and V were administered orally with CPP at a dose of 50, 100, and 200 mg/kg. After 60 min, all rats were administered with an intraperitoneal injection of acetic acid (10 ml/kg, 0.6%). After 20 min, acetic acid induced the writhing response (the abdominal min according to the procedures described [71]). The percent analgesia was calculated from the mean number of writhes in the vehicle control group (Wv) and that of the treatment group (Wt) as per formula: % analgesia = 100*[(Wv−Wt)/Wv].

### Antiarthritic Activity of CPP against adjuvant-induced established polyarthritis (AIA)

Induction of arthritis was performed according to the earlier described method [72] as modified for therapeutic schedule. AIA was induced in 6 to 8 week-old rats by a single intradermal injection of 0.1 ml Freund’s complete adjuvant (FCA) into the footpad of the right hind paw. FCA contains 0.6 mg heat-inactivated Mycobacterium tuberculosis, H37Ra (Difco Laboratory, Detroit, MI, USA) emulsified in a sterile mixture of paraffin oil, saline, and Tween 80. Arthritis was allowed to develop for the next 12 days. On day 12, animals were randomly assigned to one of the following treatment groups: Group 1: adjuvant – induced arthritis controls (AIA controls), Group 2: arthritic rats treated with celecoxib (10 mg/kg/day), and Group 3 to 5: arthritic rats treated CPP (50, 100, and 200 mg/kg/day), respectively. These groups of rats were orally treated once daily with the following treatment: Group 1: vehicle (distilled water), Group 2: celecoxib (10 mg/kg, p.o.), and Group 3 to 5: CPP (50, 100, or 200 mg/kg) from day 12 to day 21 by oral feeding needle.

The body weights of rats were recorded at the baseline (day 0), and day12 and day 21 of the study. The difference in body weight was calculated relative to that at day 0, allowing monitoring of the decrease in body weight gain associated with arthritis [73]. Swelling of hind paws was monitored by using a plethysmometer (UGO Basile Italy) at day 0 and 12 and 21 days after the FCA injection [74].

On day 21, rats were sacrificed and ulcerogenicity was investigated on stomach tissue samples. The stomach was dissected out, sectioned along the greater curvature, washed with saline, and the glandular portion was examined macroscopically for the number and size of mucosal lesions. The stomach pieces were placed in 10% buffered formaldehyde solution (Merck) and embedded in paraffin pastilles (Merck). Serial sections of 5 mm were cut and stained with Hemotaoxylin and Eosin (H&E) (Merck). Mounting of the specimen was done by using Distrene Phthalate Xylene (D.P.X.) and examined under microscope for photomicrographic analysis [75].

### Serum TNFα measurement in AIA rats

The blood samples were collected on day 0, day 13, and day 21 of post-FCA inoculation. Serum was separated and stored at −20 °C until the assay. Serum TNF-α was estimated by the BD-OptEIA kit (BD Pharmingen, U.S.A.) using the manufacturer‘s protocol. Briefly, 50 μl of ELISA diluent was added to each well followed by the TNF- α standards and samples were incubated at room temperature for 2 h. After thorough washing, 100 μl of the detection antibody (biotinylated anti-rat monoclonal TNF-α antibody) was added to each well and incubated for 1 h at room temperature. After thorough washing, 100 μl of avidinhorseradish peroxidase conjugate was added to each well and incubated for 30 min at room temperature. After incubation, 100 μl of the substrate reagent (3,3′,5,5′-tetramethylbenzidine, TMB) was added. The reaction was stopped using 1 N H2SO4 and the plates were read at 450 nm within 30 min of stopping the reaction. All results were converted to pg/ml using the standard curve on linear-log graph paper as suggested by the protocol [76].

### IL2, IL4, and IFNγ concentration measurement in ConA-stimulated lymphocytes in vitro

Three male Swiss albino mice (20–25 g) were sacrificed by cervical dislocation. The mice were dissected and spleens were removed. The spleens were then dried on a paper and used for the isolation of lymphocytes. Spleen cells were obtained by squeezing the spleen through a nylon mesh in a petriplate containing Roswell Park Memorial Institute (RPMI) medium. The hypotonic shock was given to RBCs by hypotonic NH4Cl solution. Lymphocytes were washed three times using ice-cold RPMI medium containing 10% fetal calf serum (FCS) conditioned media. All the cells were harvested in test tubes, centrifuged at 5000 rpm for 5–7 times, and resuspended in 5 ml medium. Then 25 μl of the cell suspension and 475 μl of methylene blue was mixed and cells were counted with a hemocytometer. Spleen cells were cultured with ConA and the culture supernatants were collected 72 h. after stimulation. The supernatants were used for IL-2, IL-4, and IFNγ estimation by the enzyme-linked immunosorbent assay (ELISA) kit (Pharmingen, San Jose, California, USA) as per manufacturer’s instructions [77].

Two million lymphocytes were placed in each well of a 24-well culture plate. Group distribution and incubation of cells were as follows: 1. Untreated cells served as a control, 2. ConA(10 μg/ml)-stimulated lymphocytes served as a positive control group, 3. ConA(40 μl)-stimulated lymphocytes in the presence of CPP (100 μl, 0.4% i.e. 40 μg/100 μl). The final 24-well culture plate (1 ml) concentration of three different groups was as follows: (1) Control Cells (220 μl) + FCS (120 μl) + RPMI (660 μl), (2) ConA-treated cells (220 μl) + FCS (120 μl) + ConA (40 μl) + RPMI (620 μl), and (3) CPP-treated cells (220 μl) + FCS (120 μl) + ConA (40 μl) + RPMI (520 μl) +IND 02 (100 μl). Lymphocytes were cultured for 72 h at 37°C in 2 ml culture medium in a 95% air/5% CO2 atmosphere in a CO2 incubator until assaying.

Levels of IL-2, IL4, and IFNγ were estimated by commercially available ELISA kits (BD-Opt EIA kits, BD Pharmingen, U.S.A.) using the manufacturer’s protocols. Samples were acidified with 1 N HCl and incubated at room temperature for 15 min, neutralized with 1 N NaOH, and used for the sandwich ELISA procedure. Ninety-six-well high-binding ELISA plates (BD Falcon ELISA plates) were coated with 100 μl of capture antibody (either with anti-rat IL-2 or IL-4 or IFNγ) and maintained overnight at 4 ºC. The plate was brought to room temperature the next day and was blocked by 200 μl of assay diluent and incubated for 2 h. After washing, 100 μl of standards (provided with the kit) and samples were added to each well and incubated for 1 hour. Then 100 μl of biotinylated antimouse IL-1β was used as the detection antibody. After thorough washing, 100 μl of avidin-horseradiish peroxidase conjugate was added to each well and incubated for 30 min at room temperature. After incubation, 100 μl of the substrate reagent (3,3’,5,5’-tetramethylbenzidine, TMB) was added. The reaction was stopped with 1 N H2SO4 and the absorbance was read at 450 nm in an ELISA plate reader (Biotek Instruments Inc, USA). All the results were converted to pg/ml using the standard curve on linear-log graph paper as suggested by the protocol.

### Statistical analysis

The carrageenan-induced rat paw edema experiment (paw volume), FCA-induced established polyarthritis, AIA model (body weight, paw volume, and TNFα levels), and ConA-stimulated lymphocytes in vitro experiment (IL2, IL-4 and IFN-γlevels) were analysed by separate two-way ANOVA followed by Bonferroni post-tests. The data obtained in the cotton pellet granuloma experiment (mean weight of cotton pellets) and acetic acid-induced writhing model in rats (mean number of writhes) were analysed by separate one-way ANOVA followed by Dunnett’s t-test. P < 0.05 was considered significant.

## Figures and Tables

**Tab. 1:** Effect of CPP against carrageenan-induced rat paw edema in rats

**Treatment (dose, mg/kg, route)**	**Mean Paw Volume (mL.) ± SEM**				**PWT in Randall-Selitto assay at 3 h (g ± SEM) (% increase)**
	**0 h**	**1 h**	**2 h**	**3 h**	
Vehicle control	2.60 ± 0.07	3.65 ± 0.21	4.81 ± 0.21	5.23 ± 0.13	66.00 ± 7.8
Celecoxib (10, p.o.)	2.75 ± 0.07	3.55 ± 0.15 (23.44)	4.43 ± 0.17 (24.04)	4.18^c^ ± 0.29 (45.79)	90.00^c^ ± 7.9 (36.4)
CPP (50, p.o.)	2.79 ± 0.07	3.70 ± 0.19 (12.76)	4.72 ± 0.17 (12.81)	4.84^b^ ± 0.37 (21.83)	74.3 ± 4.08 (12.63)
CPP (100, p.o.)	2.72 ± 0.10	3.24 ± 0.13 (12.28)	4.28 ± 0.05 (29.54)	4.48 ± 0.09 (33.19)	75.00^a^ ± 4.14 (16.66)
CPP (200, p.o.)	2.47± 0.10	3.30 ± 0.10 (21.05)	3.91^c^± 0.14 (37.27)	4.05^c^± 0.03 (39.97)	81.0^c^ ± 0.97 (20. 73)

n=6, Data is represented as Mean paw volume (ml.) ± SEM. and was analyzed by Two-way repeated measures ANOVA followed by Bonferroni posttests. Significance represented as

a P < 0.05,

b P < 0.01,

c P < 0.001 as compared to vehicle at respective time point. PWT…Paw withdrawal Threshold.

**Tab. 2.** Effect of CPP in cotton pellet granuloma- and acetic acid-induced writhing model in rats

**Treatment**	**Mean weight of cotton pellet granuloma ± S.E.M. (% Inhibition)**	**Mean number of writhes against acetic acid (% analgesia)**
Vehicle control	44.67 ± 1.60	29.17 ± 0.48
Celecoxib (10, p.o.)	25.23 ± 0.47^c^ (43.51)	13.83 ± 0.60^c^ (52.59)
CPP (50, p.o.)	32.47 ± 0.49^c^ (27.31)	25.33 ± 1.05 (13.16)
CPP (100, p.o.)	29.30 ± 0.81^c^ (34.40)	23.83 ± 1.30 (18.31)
CPP (200, p.o.)	27.28 ± 1.25^c^ (38.92)	22.00 ± 1.53^a^ (24.58)

n=6, Data is represented as Mean value ± SEM. and was analyzed separately for each parameter by One-way ANOVA followed by Dunnett’s t test. Significance represented as

a P < 0.05,

c P < 0.001 as compared to vehicle control.

**Tab. 3.** Effect of CPP on Body weight, Paw edema, and serum TNFα levels at baseline, Day-12 and Day-21 post-sensitization in FCA-induced established polyarthritis (AIA model)

**Parameter**	**Day**	**Mean Parameter value (dose in mg/kg)**				
		**AIA**	**Celecoxib (10)**	**CPP (50)**	**CPP (100)**	**CPP (200)**
Body weight (g)	0	171.00 ± 1.67	170.17 ± 1.99	170.17 ± 1.99	173.33 ± 3.02	170.33 ± 4.29
	12	141.00^a^ ± 2.16	159.17^a^ ± 2.75	159.17^a^ ± 2.75	162.50^a^ ± 1.84	158.00^a^ ± 2.19
	21	136.33 ± 2.40	188.33^b^ ± 3.00	188.33^b^ ± 3.00	195.17^b^ ± 1.60	197.67^b^ ± 3.38

Paw Volume (ml) – Injected – Left	0	2.67 ± 0.14	2.47 ± 0.17	2.56 ± 0.13	2.13 ± 0.08	2.47 ± 0.15
	12	5.75^a^ ± 0.09	5.28^a^ ± 0.16	5.59^a^ ± 0.16	5.18^a^ ± 0.18	5.25^a^ ± 0.85
	21	5.65 ± 0.09	3.18^b^ ± 0.06	4.24^b^ ± 0.26	3.31^b^ ± 0.17	3.40^b^ ± 0.62
	
Paw Volume (ml) – Contralateral – Right	0	2.16 ± 0.15	1.99 ± 0.13	1.96 ± 0.14	2.18 ± 0.03	2.03 ± 0.41
	12	3.44^a^ ± 0.11	3.42^a^ ± 0.09	3.42^a^ ± 0.29	3.65^a^ ± 0.08	3.60^a^ ± 0.59
	21	3.90 ± 0.16	2.81 ± 0.14	3.78 ± 0.45	3.15 ± 0.08	2.26^b^ ± 0.05

TNFα levels (pg/ml)	0	20.05 ± 0.25	28.86 ± 0.48	–	–	33.53 ± 0.42
	12	49.63^a^ ± 2.53	47.82^a^ ± 2.94	–	–	47.45^a^ ± 2.03
	21	52.58 ± 2.44	33.72^b^ ± 2.70	–	-–	37.40^b^ ± 2.83

n=6, Data is represented as Mean parameter value ± SEM. The data for each parameter was separately analyzed by Two-way repeated measures ANOVA followed by Bonferroni posttests. Significance represented as

a P < 0.001 as compared with baseline (day-0) values of same treatment group.

b P < 0.001 as compared to Day-12 values of same treatment group.

**Tab. 4.** Effect of CPP on IL2, IL-4, and IFN-γ in ConA-stimulated lymphocytes *in vitro*

**Treatment**	**Cytokine concentration ± S.E.M. (pg/ml)**		
	**IL-2**	**IL-4**	**IFNγ**
Control	1.94 ± 0.24	25.02 ± 1.03	20.41 ± 0.54
ConA (40 μl)	589.00 ± 8.8^a^	114.75 ± 3.89^a^	277.74 ± 7.26^a^
CPP (100 μl, 0.4%)	10.27 ± 0.26^b^	41.22 ± 1.90^b^	21.98 ± 1.03^b^

Each reading is mean of triplicate readings obtained from ELISA assay. Data was analyzed by Two-way ANOVA followed by Bonferroni posttests.

a P < 0.001 as compared with Control^,^

b P<0.001 as compared with ConA treated group test.
